# Age-related changes in factors associated with delayed extubation after general anesthesia: a retrospective study

**DOI:** 10.1186/s40981-020-00325-8

**Published:** 2020-03-13

**Authors:** Naoya Kobayashi, Toshihiro Wagatsuma, Takuya Shiga, Hiroaki Toyama, Yutaka Ejima, Masanori Yamauchi

**Affiliations:** Department of Anesthesiology and Perioperative Medicine, Tohoku University Graduate School of Medicine, 2-1 Seiryomachi, Aoba, Sendai, Miyagi 980-8575 Japan

To the Editor

The number of older adults who undergo surgical procedures has increased in recent years, especially in developed countries [1, 2]. Early recovery is a primary goal when providing general anesthesia to older adults. Since previous studies revealed that shorter extubation times allow for early recovery and are associated with reduced pharmacological and perioperative complications [3–7], the extubation time (defined as the time elapsed from cessation of sedative drugs to extubation) has become a topic of interest to surgeons and anesthesia care providers. Although delayed extubation occurs more frequently in older than younger patients, age-related changes in factors influencing extubation times remain relatively unexplored. Therefore, we conducted a large-scale retrospective analysis of the association between extubation times and covariates such as comorbidities, sedative and analgesic agents, operation methods, and intraoperative management in both older and younger patients.

We analyzed data from 9514 patients who underwent surgical procedures under general anesthesia in a university hospital between 2011 and 2015. Ethical approval was obtained from the Ethics Committee of Tohoku University Graduate School of Medicine (reference number: 2015-1-533). The requirement for informed consent was waived due to the retrospective nature of the study. Patients were divided into two groups according to age: one group comprised of patients aged less than 65 years (< 65 years) and the other group comprised of patients aged 65 years or more (≥ 65 years). The extubation adopted the following commonly adopted criteria [8]: (1) consciousness: open eyes, fate response; (2) respiration: spontaneous breathing, appropriate respiratory rate (< 25/min, > 8/min), tidal volume (> 5 mL × body weight [kg]), No tracheal secretions, PaO_2_/F_I_O_2_ > 300 mmHg; (3) stable circulatory dynamics; (4) recovery of muscle strength: hold head for 5 s or more, hold/open hand, TOF ratio 0.9 or more; (5) no abnormalities on chest X-ray before extubation. Because many anesthesiologists regard extubation times over 15 min as indicative of poor recovery [9], the time from the discontinuation of sedative agents to extubation was regarded as the primary endpoint of the present study.

We performed logistic regression analysis to assess the association of various factors with delayed extubation. In both age groups, delayed extubation was associated with the male sex (< 65 years: *P* < 0.001; ≥ 65 years: *P* < 0.001), brain surgery (< 65 years: *P* = 0.004; ≥ 65 years: *P* < 0.001), and selection of sedative agents (< 65 years: *P* < 0.001; ≥ 65 years: *P* = 0.006). In the < 65 years group, delayed extubation was also associated with emergency surgery (*P* = 0.048). In the ≥ 65 years group, delayed extubation was associated with age (*P* < 0.001), cervical surgery (*P* < 0.001), abdominal surgery (*P* = 0.032), and orthopedic surgery (*P* = 0.028). Other comorbidities, intraoperative fluid balance, bleeding, and operation time were not associated with delayed extubation (Fig. 1). As a result, the influence of the anesthesiologist’s decision on delayed extubation was greater in the elderly. In addition, our findings suggest that extubation in patients who were 65 years or older was more delayed during cervical surgery.

**Fig. 1:**
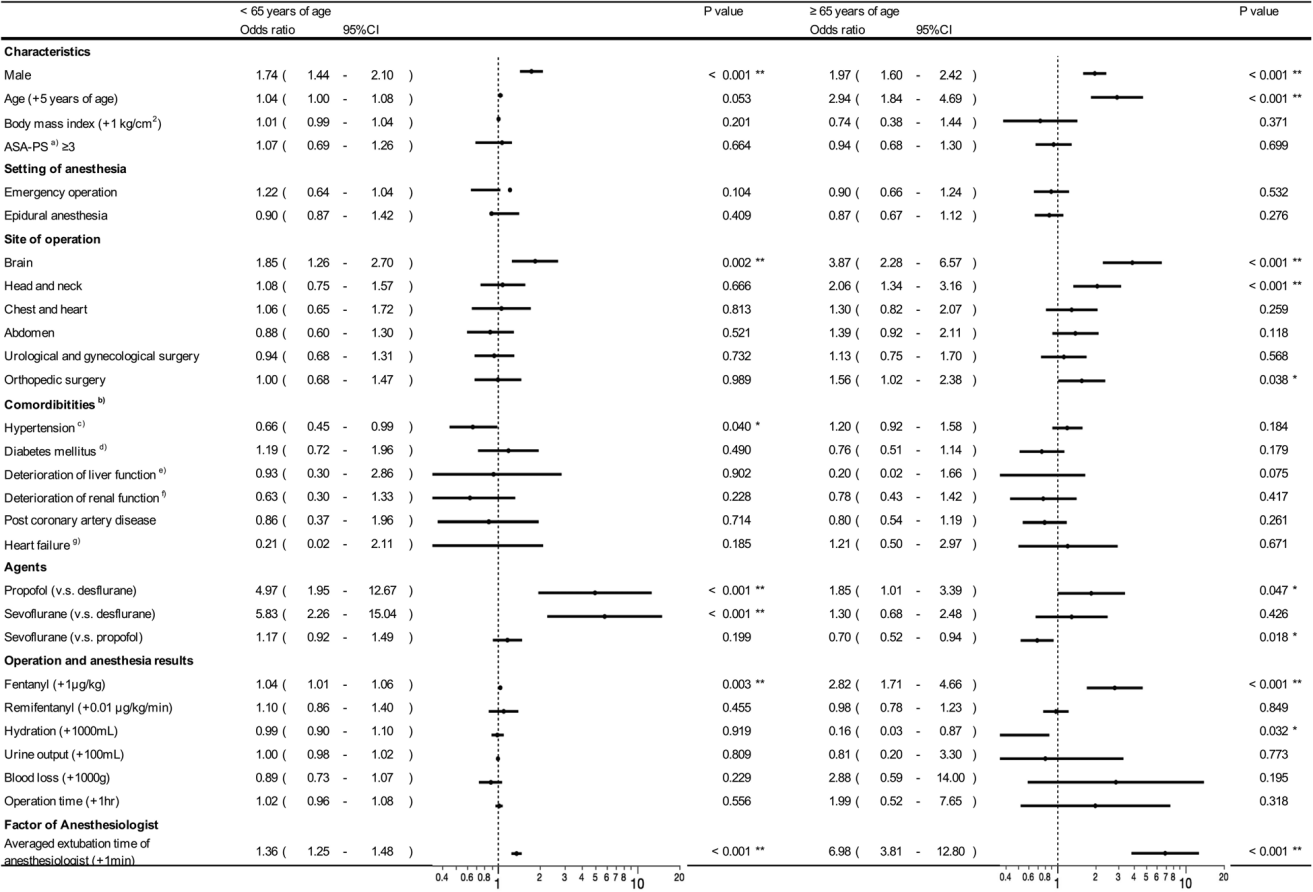
Association between various clinical factors and delayed extubation (> 15 min). Statistical analysis was performed using a multiple logistic regression model. For quantitative data, odds ratios are described per value in parentheses. *P < 0.05, **P < 0.01. ^a^American Society of Anesthesiologists physical status. ^b^Comorbidities were extracted from the anesthesia ledger system (JSA-PIMS from Japanese Society of Anesthesiologists). ^c^Hypertension was defined as systolic blood pressure ≥ 140 mmHg, diastolic blood pressure as ≥ 90 mmHg, or the use of medications for blood pressure control. ^d^Diabetes mellitus was defined as the use of oral hypoglycemic agents or insulin. ^e^Deterioration of liver function was defined as AST >32, ALT >37, or the presence of cirrhosis. ^f^Deterioration of renal function was defined as estimated GFR < 60 mL/min/1.73m^2^. ^g^Heart failure was defined based on Framingham criteria [10].

In conclusion, our findings indicate that the influence of the anesthesiologist’s decision on delayed extubation was greater in patients ≥ 65 years of age. In addition, our findings suggest that extubation during cervical surgery was more delayed in patients who were 65 years or older.

## Data Availability

Not applicable.
